# U2AF2-SNORA68 promotes triple-negative breast cancer stemness through the translocation of RPL23 from nucleoplasm to nucleolus and c-Myc expression

**DOI:** 10.1186/s13058-024-01817-6

**Published:** 2024-04-09

**Authors:** Wenrong Zhang, Xinyue Song, Zining Jin, Yiqi Zhang, Shan Li, Feng Jin, Ang Zheng

**Affiliations:** 1 Department of Breast Surgery, The First Hospital of China Medical University, Shenyang, Liaoning Province China; 2 Department of Pharmacology, Liaoning Province Key Laboratory of Molecular Targeted Antitumour Drug Development and Evaluation, China Medical University, Shenyang, Liaoning Province China; 3 Department of Breast Surgery, The First Hospital of Jinzhou Medical University, Shenyang, Liaoning Province China; 4 Department of General Surgery, Beijing Chao-Yang Hospital, Capital Medical University, Beijing, China

**Keywords:** Triple-negative breast cancer, stemness, SNORA68, RPL23, Translocation to nucleolus, U2AF2

## Abstract

**Background:**

Small nucleolar RNAs (snoRNAs) play key roles in ribosome biosynthesis. However, the mechanism by which snoRNAs regulate cancer stemness remains to be fully elucidated.

**Methods:**

SNORA68 expression was evaluated in breast cancer tissues by in situ hybridization and qRT‒PCR. Proliferation, migration, apoptosis and stemness analyses were used to determine the role of SNORA68 in carcinogenesis and stemness maintenance. Mechanistically, RNA pull-down, RNA immunoprecipitation (RIP), cell fractionation and coimmunoprecipitation assays were conducted.

**Results:**

SNORA68 exhibited high expression in triple-negative breast cancer (TNBC) and was significantly correlated with tumor size (*P* = 0.048), ki-67 level (*P* = 0.037), and TNM stage (*P* = 0.015). The plasma SNORA68 concentration was significantly lower in patients who achieved clinical benefit. The SNORA68-high patients had significantly shorter disease-free survival (DFS) (*P* = 0.036). Functionally, SNORA68 was found to promote the cell stemness and carcinogenesis of TNBC in vitro and in vivo. Furthermore, elevated SNORA68 expression led to increased nucleolar RPL23 expression and retained RPL23 in the nucleolus by binding U2AF2. RPL23 in the nucleolus subsequently upregulated c-Myc expression. This pathway was validated using a xenograft model.

**Conclusion:**

U2AF2-SNORA68 promotes TNBC stemness by retaining RPL23 in the nucleolus and increasing c-Myc expression, which provides new insight into the regulatory mechanism of stemness.

**Supplementary Information:**

The online version contains supplementary material available at 10.1186/s13058-024-01817-6.

## Background

At present, breast cancer is the most common malignant tumor worldwide [1, 2]. Triple-negative breast cancer (TNBC) is characterized by a lack of estrogen receptor (ER), progesterone receptor (PR) and HER2 expression. Breast cancer is a biologically aggressive tumor characterized by highly proliferative and metastatic cancer cells, which, together with limited treatment options, leads to the poorest prognosis and highest susceptibility to recurrence among breast cancer subtypes. However, clinicians still lack tumor-specific targeted therapies for TNBC. Filtering novel biomarkers and therapeutic targets is considered to be an effective way to improve TNBC treatment strategies.

Small nucleolar RNAs (snoRNAs) are a type of noncoding RNA located in the nucleolus and have a length of 60–300 nucleotides. Based on their sequences and structure, snoRNAs are mainly classified as box C/D SNORD and box H/ACA SNORA. SnoRNAs are involved in processes such as rRNA processing, RNA alternative splicing, translation regulation, and oxidative stress response [3–5]. Accumulating evidence has indicated the role of snoRNAs in the pathology of various cancers. As an epigenetic regulator, abnormally expressed snoRNAs can activate or inhibit cancer-related signalling pathways by regulating target genes and altering cell biological behaviors, thereby regulating the proliferation, self-renewal and metastasis of cancer cells and affecting tumorigenesis [6–8]. For example, SNORD33, SNORD66 and SNORD76 are highly expressed in the tissues and plasma of lung cancer patients and can be used as potential prognostic markers [9].

In breast cancer, a combination of snoRNAs has been confirmed to be associated with clinicopathological features and to show prognostic and predictive potential [10]. Moreover, SNORA71 promoted epithelial–mesenchymal transition in breast cancer cells [11]. SNORA51 enhances breast cancer stem cell-like properties [12]. Some studies have reported that snoRNAs are abnormally expressed in TNBC and regulate the occurrence and development of TNBC [13, 14]. The plasma SNORD33 concentration is an indicator of platinum-based chemotherapy sensitivity among metastatic TNBC patients, suggesting that snoRNAs have the potential to serve as a predictive marker for the efficacy of chemotherapy in TNBC patients [15].

In this study, large-scale screening of the differentially expressed snoRNAs between MCF-7 cells and MDA-MB-231 (TNBC) cells was performed via a snoRNA microarray. After bioinformatics analysis, SNORA68 was predicted to be closely related to the malignant phenotype of breast cancer. SNORA68, located at 19p13.11, is 133 bp in length and possesses one exon. To date, the function of SNORA68 in TNBC has been completely unknown.

Notably, snoRNAs are widely involved in the regulation of ribosome biosynthesis and thus affect the progression of malignant tumors [16]. Abnormal elevation or defects in ribosome biosynthesis can increase the risk of malignant tumors [17]. Both snoRNAs and ribosomal proteins (RPs) play important roles in cancer cells. Overexpression of SNORA18L5 caused the ribosomal proteins RPL5 and RPL11 to stay in the nucleolus and prevented them from binding to MDM2, which resulted in increased MDM2-mediated ubiquitination and degradation of p53 [18]. Therefore, further research is needed to explore whether SNORA68 influences breast cancer stemness by interacting with RPs.

To date, there are few reports about snoRNAs related to breast cancer stemness. Herein, we screened SNORNAs related to TNBC and revealed that SNORA68 plays an important role in the stemness regulation of TNBC cells. Our study provides new insight into the regulatory mechanism of TNBC stemness.

## Experimental procedures

### Patients and clinical samples

Fresh TNBC tissues and paired adjacent normal tissues (n = 7 pairs) were collected from the First Hospital of China Medical University from June to July 2022. The inclusion criteria were as follows: I. TNBC patients who were receiving NACT. II. The available tissues were subjected to core needle biopsy before NACT. III. Complete records with evaluation results every two cycles were obtained. Fresh tissues were snap frozen in liquid nitrogen immediately after biopsy and stored. According to the Response Evaluation Criteria in Solid Tumors (RECIST1.0), the clinical response to NACT included complete response (CR), partial response (PR), progressive disease (PD) and stable disease (SD). Clinical benefit (CB) was defined as CR, PR or SD > 6 months. Blood samples were obtained 5 days before the first NACT and collected from February to July 2023. The samples were centrifuged at 1500 × g for 10 min, and the liquid component (plasma) was transferred to RNase-free tubes. The plasma was stored at − 80 °C for further experiments.

Paraffin-embedded specimens (n = 80 patients) were obtained from patients hospitalized between September 2012 and December 2013. Patients were diagnosed with TNBC, and paraffin sections of primary breast lesions were collected. The follow-up information was collected from patients or immediate family members. This study was approved by the Ethics Committee of China Medical University (Approval number: AF-SOP- 07-1.1-01).

## Cell culture and transfection

Human breast cancer cell lines, including MCF-7, T47D, SKBR3, MDA-MB-231, BT-549, MDA-MB-453, MDA-MB-468 and HCC1937, as well as the immortalized human breast cell line MCF-10A, were purchased from the Cell Bank of Type Culture Collection (CBTCC, Shanghai, China). All cell lines were tested for mycoplasma, DNA fingerprinting, isozymes and cell viability by a third-party biological service organization (GeneCreate Biology Co., Ltd., Wuhan, China). MCF-7 cells were cultured in MEM supplemented with 10% fetal bovine serum (FBS) and 10 μg/mL insulin, while BT-549 cells were cultured in RPMI-1640 medium supplemented with FBS at concentrations of 10% and 10 μg/mL insulin. T47D and HCC1937 cells were cultured in a 37 °C humidified incubator with 5% CO_2_ in RPMI-1640 medium. SKBR3 cells were cultured in McCoy's 5A medium, while L15 Leibovitz's medium supplemented with FBS was used to culture MDA-MB-231, MDA-MB-453, and MDA-MB-468 cells.

ASO-SNORA68 and ASO-NC were obtained from RiboBio Co., Ltd. (Guangzhou, China). Cells were transfected at 40% confluence using X-tremeGENE siRNA Transfection Reagent (Catalogue#: 04476093001, Roche Diagnostics GmbH, Mannheim, Germany) with a final ASO concentration of 60 nM. The target sequence of ASO-h-SNORA68_001 was AATTTGGAGGTTCCACAACT.

## Cell functional omics

For the cell proliferation assay, equal numbers of cells were cultured in a 96-well plate. At the indicated time points, the viability of the cells was determined using a Cell Counting Kit 8 (CCK-8, Wuhan Promoter Biological Co., Ltd., #P5090) and measured at a wavelength of 450 nm with an enzyme-linked immunosorbent assay plate reader (Bio-Tek Elx 800, USA). Five replicates were performed for each group to measure the optical density (OD) values.

For the colony formation assays, MDA-MB-231 and BT-549 cells (2 × 10^3^) were seeded into 6 cm petri dishes and cultured at 37 °C in a humidified atmosphere containing 5% CO_2_. After incubation for 14 days, the cells were washed with PBS, fixed in paraformaldehyde for 15 min, and stained with a solution of crystal violet (0.5%) for another 15 min.

For the apoptosis assay, TNBC cells (1 × 10^6^) were resuspended in binding buffer and stained using an Annexin V-FITC/PI apoptosis detection kit (BD Biosciences, #556,547) following the manufacturer’s instructions. Flow cytometry (BD FACS Calibur, BD Biosciences, San Diego, CA, USA) was used to detect cell apoptosis.

For the cell migration assay, cells starved in medium without serum for 4 h were used. Serum-free medium (200 μL with 1 × 10^4^ cells) was added to the upper chamber, and 600 μL of medium supplemented with 10% serum was added to the lower chamber for each group. After incubation for 48 h, three random visual fields of the fixed and stained cells in each chamber were counted.

A sphere formation assay was conducted as previously described [19]. Briefly, suspensions of MDA-MB-231 and BT-549 cells (1 × 10^3^/well) were plated in ultralow adhesion plates (Corning, Kraemer, CA). The cells were cultured in 2 ml of serum-free DMEM-F12 supplemented with 10 μg/L bFGF (Sino Biological, Beijing, China), 20 μg/L EGF (Sino Biological, Beijing, China) and 2% B27 (Absin, Shanghai, China).

## Cell fractionation

Briefly, 1 × 10^7^ cells were resuspended in 500 µL of hypotonic buffer (10 mM HEPES [pH 7.9], 10 mM KCl, 1.5 mM MgCl_2_ and 0.5 mM DTT). The suspension was thoroughly vortexed and vibrated for 10–30 min at 4 °C. Following centrifugation (2,000 × g) for a period of 5 min, the resulting supernatant was collected as the cytoplasmic fraction. The obtained pellet was then resuspended in S1 buffer (0.25 M sucrose and 10 mM MgCl_2_), layered onto 300 µL of S2 buffer (0.35 M sucrose and 0.5 mM MgCl_2_) and subjected to centrifugation (1,500 × g) for another five min. The pellet was resuspended in S2 buffer to serve as the nuclear fraction. The nuclear fraction underwent sonication for two seconds followed by three second intervals over a span of six to seven min before being overlaid onto S3 buffer (0.88 M sucrose, 0.05 mM MgCl_2_). Centrifugation at a speed of approximately three thousand times gravity (3000 × g) for 10 min allowed collection of the resulting supernatant as the nucleoplasmic fraction, while the obtained pellet served as the nucleolar fraction after being resuspended in RIPA buffer solution. The supernatant was collected as the nucleoplasmic fraction. The pellet was resuspended in RIPA buffer to serve as the nucleolar fraction. The fractions were subsequently resolved through SDS‒PAGE analysis followed by immunoblotting (IB).

## Immunofluorescence (IF)

Cells were cultured on coverslips in 12-well plates, fixed with 4% paraformaldehyde for 15 min at room temperature, and then permeabilized with 0.5% Triton X-100 for 10 min. After blocking with 5% bovine serum albumin for 1 h, the cells were incubated overnight at 4 °C with the indicated primary antibodies. Subsequently, the appropriate secondary antibodies (FITC or DyLight549-conjugated goat anti-mouse IgG and DyLight549 or DyLight649-conjugated goat anti-rabbit IgG) were incubated with the cells for 1 h at 37 °C. DAPI was used to counterstain the nuclei. Confocal laser-scanning microscopy on a Nikon Digital ECLIPSE C1 system (Nikon Corporation) was utilized to capture images.

## Fluorescence in situ hybridization (FISH) and IF

A combination of FISH and IF was performed. Before the IF assay, prehybridization solution was added to the cells at 37 °C for 1 h. Hybridization solution containing the SNORA68 probe was added, and the cells were hybridized overnight. Then, the cells were incubated with the primary antibody and secondary antibody (Additional file 1: Supplementary Table S1). The membranes were incubated with primary antibody overnight at 4 °C and then washed with PBS 3 times. The corresponding secondary antibody was added dropwise, and the cells were incubated at room temperature for 50 min and then washed with PBS 3 times. DAPI dye was used to stain the nuclei for 8 min in the dark. Images were captured by a fluorescence microscope.

## RNA pull-down assay

TNBC cells (4 × 10^7^) were lysed in 1 mL of RIP buffer, and cell lysates were cleared by centrifugation at 13,000 rpm for 10 min at 4 °C. Biotinylated RNA probes against SNORA68 (100 pmol) were then incubated with cell lysates for 4 h at 37 °C. Following this step, 50 μl of washed streptavidin magnetic beads (Invitrogen) was added to each binding reaction and further incubated for one hour at room temperature. The beads were collected after washing five times with RIP washing buffer. SDS‒PAGE or mass spectrometry analysis was used for analysis.

## RNA-binding protein immunoprecipitation (RIP)

RIP was performed using a Magna RIP™ RIP Kit (Millipore, Bedford, MA, USA) based on the manufacturer’s protocol [20]. In brief, TNBC cells (1 × 10^7^) were lysed in 200 μl of RIP buffer, and cell lysates were removed by centrifugation at 13,000 rpm for 10 min at 4 °C. The cleared lysates were set aside as input and stored at − 80 °C. Then, the lysates were incubated with beads precoated with antibodies overnight at 4 °C. The beads were washed 7 times with washing buffer. The beads were resuspended in 2 × loading buffer to elute proteins or resuspended in TRIzol extract RNA after being treated with DNase and proteinase K. SNORA68 expression was measured by qRT‒PCR after reverse transcription.

## Coimmunoprecipitation (Co-IP)

A total of 1 × 10^7^ cells were harvested and lysed in 500 μl of IP buffer (50 mM Tris–HCl, 150 mM NaCl, 1% Triton X-100, 1 mM EDTA, 10% glycerol, and protease inhibitor cocktail, pH 7.4). Then, the cell lysates were cleared by centrifugation at 12,000 rpm for 20 min at 4 °C. After being precleared with protein G-conjugated agarose (GE Healthcare Life Sciences) for 4 h, the cell lysates were incubated with specific antibodies overnight at 4 °C. Then, the cell lysates were immunoprecipitated with protein G-conjugated agarose for 4 h at 4 °C and washed with IP-wash buffer (50 mM Tris–Cl, 300 mM NaCl, 1% Triton X-100, 1 mM EDTA, pH 7.4).

## Limiting dilution assay (LDA)

For the in vitro LDA experiment, the indicated numbers of TNBC cells were seeded at a density of 256, 128, 64, 32, 16, 8, 4, 2, or 1 per 96-well ultralow attachment plate and cultured in DMEM-F12 supplemented with 10 μg/L bFGF, 20 μg/L EGF and 2% B27. The colonies were cultured for 2 weeks. The frequency of TNBC stem/initiating cells was analysed by ELDA software (http://bioinf.wehi.edu. au/software/elda/).

## Bioinformatics analysis

This study used SNORic (http://bioinfo.life.hust.edu.cn/SNORic/) to analyse the relationship between SNORA68 expression and survival and ENCORI (https://starbase.sysu.edu.cn/) to predict the proteins that interact with SNORA68. Using the RPISeq database (http://pridb.gdcb.iastate.edu/RPISeq/), this study analysed whether there was a possibility of interaction between the input proteins and RNA sequences.

The edgR package, which was downloaded from TCGA-BRCA (https://cancergenome.nih.gov/) in the R environment, was used to normalize gene expression. Gene set enrichment analysis (GSEA) was performed (http://www.broadinstitute.org/gsea/index.jsp) [21]. The samples in the TCGA-BRCA cohort were divided into SNORA68-high and SNORA68-low groups according to the median expression. The inclusion criteria were a normalized p < 0.05 and a false discovery rate (FDR) < 25%. The significantly enriched pathways were chosen according to the normalized enrichment score (NES). KEGG functional enrichment analysis was used to detect the biological functions and pathways influenced by the DEGs in the Database for Annotation Visualization and Integrated Discovery (DAVID) (https://david.ncifcrf.gov/).

Molecular Operating Environment (MOE) (Chemical Computing Group, Montreal, QC, Canada) RNA‒Protein Dock was used for SNORA68 and U2AF2 interaction. The crystal structure of SNORA68 was predicted by 3dRNA (http://biophy.hust.edu.cn/new/3dRNA). The crystal structure of U2AF2 was selected from UniProt (https://www.uniprot.org/), and AF-P26368-F1 predicted by Alphafold (accessed on 20 Sep 2023) was used. One hundred poses of RNA and protein were performed, and we chose the lowest energy pose representing the strongest binding [21].

## RNA extraction and quantitative real-time polymerase chain reaction (qRT‒PCR)

RNA extraction and qRT‒PCR were performed as previously described [19]. In addition, snoRNA extraction from plasma and quantification of plasma SNORA68 expression by qRT‒PCR were performed according to a previously reported protocol [15]. The primer sequences are presented in Additional file 1: Supplementary Table S2.

## Western blot

Western blot was performed as previously described [22]. The NE-PER™ Nuclear and Cytoplasmic Extraction Reagents Kit (Thermo Scientific, USA) was used to isolate nuclear and cytoplasmic fractions. All antibodies used are shown in Additional file 1: Supplementary Table S1.

## In situ hybridization (ISH) and immunohistochemistry (IHC)

ISH was performed as previously described [19]. According to the protocol of the snoRNA ISH Kit (Boster), SNORA68 expression was estimated by double-score semiquantitative analysis. At least ten fields of each slide were examined, and 100 cells were observed during each examination at 400 × magnification. The staining intensity was recorded as 0 (negative), 1 (light), 2 (medium) or 3 (deep). The percentages of positive cells were scored as 0 (< 5%), 1 (6–25%), 2 (26–50%), 3 (51–75%) or 4 (> 76%). The final ISH staining score was determined by multiplying the two scores. Patients were categorized into two groups: SNORA68-high (score > 3) and SNORA68-low (score ≤ 3). An Ultrasensitive™ S-P Kit (Maixin-Bio, China) was used. All antibodies used are shown in Additional file 1: Supplementary Table S1.

## Xenograft model

A total of 1.0 × 10^6^ BT-549 cells stably transfected with oe-SNORA68 and sh-U2AF2 were suspended in 100 μl of PBS and injected into each mammary fat pad of 3/4-week-old female BALB/c (nu/nu) mice (Hua Fukang Biological Technologies Inc., Beijing). The mice were randomized into four groups (n = 6 per group). Once the tumors had formed, the tumor diameter and weight were measured every other day. The tumors were removed and weighed until the largest tumor was close to 2 cm in size. All mice were bred under pathogen-free conditions in the Animal Center.

## Statistical analysis

GraphPad Prism 8.0 (La Jolla, CA, USA) and SPSS 24.0 (Chicago, IL, USA) were used to conduct the statistical analyses. The results are presented as the mean ± standard deviation for at least three independent experiments. Student’s independent t test was used to compare multiple groups. The relationships between SNORA68 and clinicopathological factors were examined by Pearson chi-square tests. Probability values less than 0.05 were regarded as statistically significant.

## Results

### SNORA68 was highly expressed in TNBC

**Fig. 1:**
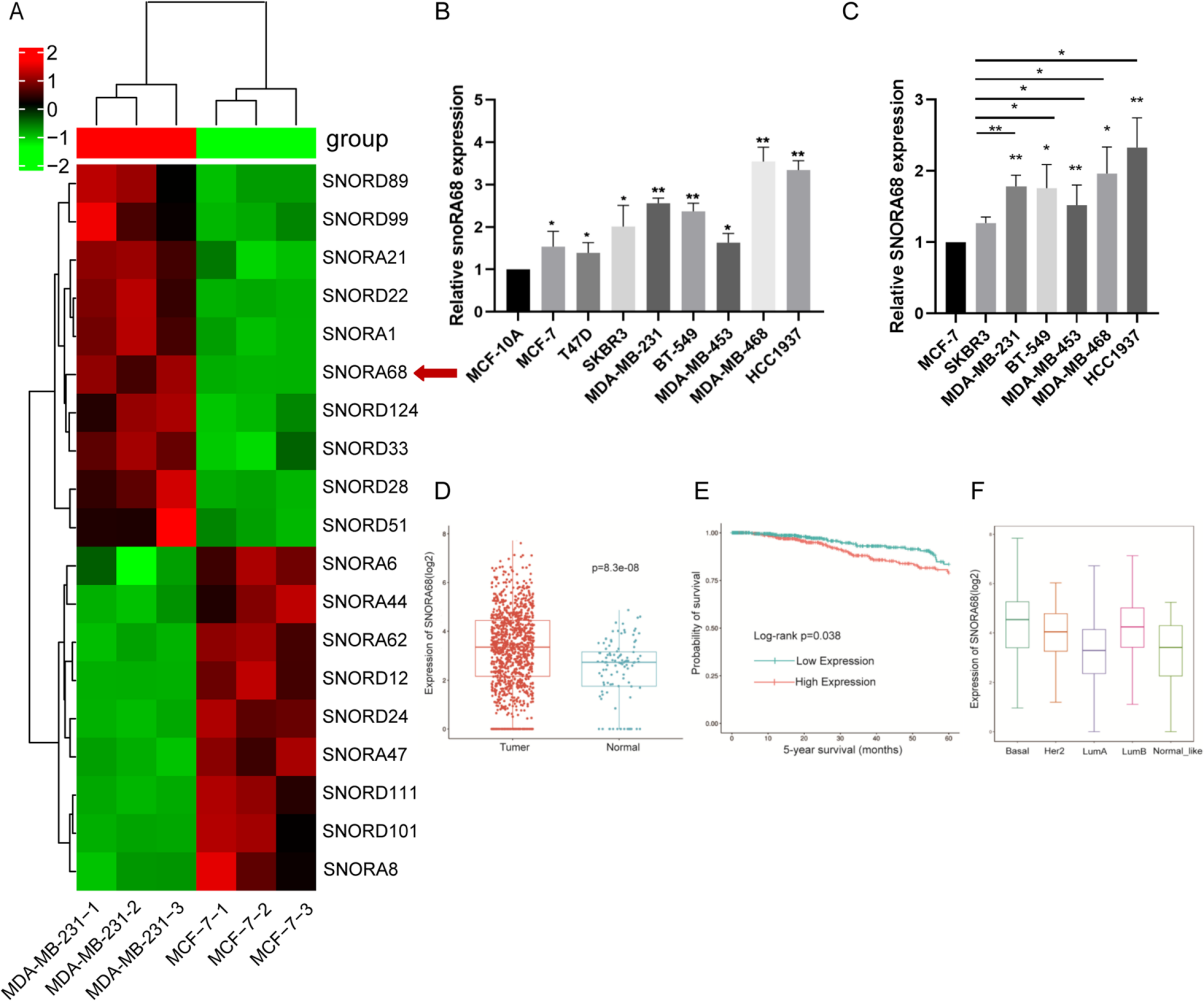
SNORA68 is upregulated in TNBC cells and is related to TNBC. **A** The heatmap shows that the expression level of SNORA68 was greater in MDA-MB-231 cells than in MCF-7 cells, as detected by snoRNA sequencing. **B** qRT‒PCR was used to detect the expression of SNORA68 in human normal breast epithelial cells and breast cancer cells of different subtypes. **C** qRT-PCR was used to detect the expression of SNORA68 between MCF-7, SKBR3 and TNBC cells. **D** The expression of SNORA68 in breast cancer was analysed via the SNORic database. **E** The relationship between SNORA68 and survival among breast cancer patients was analysed via the SNORic database. **F** SNORic image shows the differential expression of SNORA68 in subtypes of breast cancer.* *P* < 0.05, ** P < 0.01

To identify snoRNAs aberrantly expressed in TNBC, we performed snoRNA sequencing (Aksomics, Shanghai, China) to analyse the differential expression of snoRNAs between MCF-7 and MDA-MB-231 cells. The heatmap shows the genes with the most significant differences (Fig. 1A). SNORA68 was chosen after several of the top candidates were tested in cell lines because SNORA68 was stably and significantly more highly expressed in all TNBC cell lines than in luminal cell lines. For example, there was no significant difference in SNORA21 expression between MCF-10A and MCF-7 cells. SNORA21 was expressed at lower levels in BT-549 and MDA-MB-468 cells than in MCF-7 cells (Additional file 1: Supplementary Figure S1). Furthermore, we used qRT‒PCR to analyse SNORA68 expression in breast cancer cells and found that the expression of SNORA68 was significantly upregulated in TNBC cell lines (MDA-MB-231, BT-549, MDA-MB-453, MDA-MB-468 and HCC1937) compared with that in other types of breast cancer cell lines (MCF-7 and SKBR3) and a human breast epithelial cell line (MCF-10A) (Fig. 1B, C). We used the SNORic database to analyse SNORA68 expression in the whole breast cancer population and found that SNORA68 was upregulated in breast cancer (Fig. 1D). SNORA68 was associated with unfavourable survival in patients with breast cancer (Fig. 1E). SNORA68 had the highest expression in TNBC (basal type) (Fig. 1F). As a result of the limitations of bioinformatics, the expression levels of SNORA68 cannot be analysed specifically for the TNBC subtype. Therefore, we explored the correlation between SNORA68 expression and TNBC subtypes in our clinical samples.

## SNORA68, a clinically relevant snoRNA, is correlated with TNBC

**Fig. 2:**
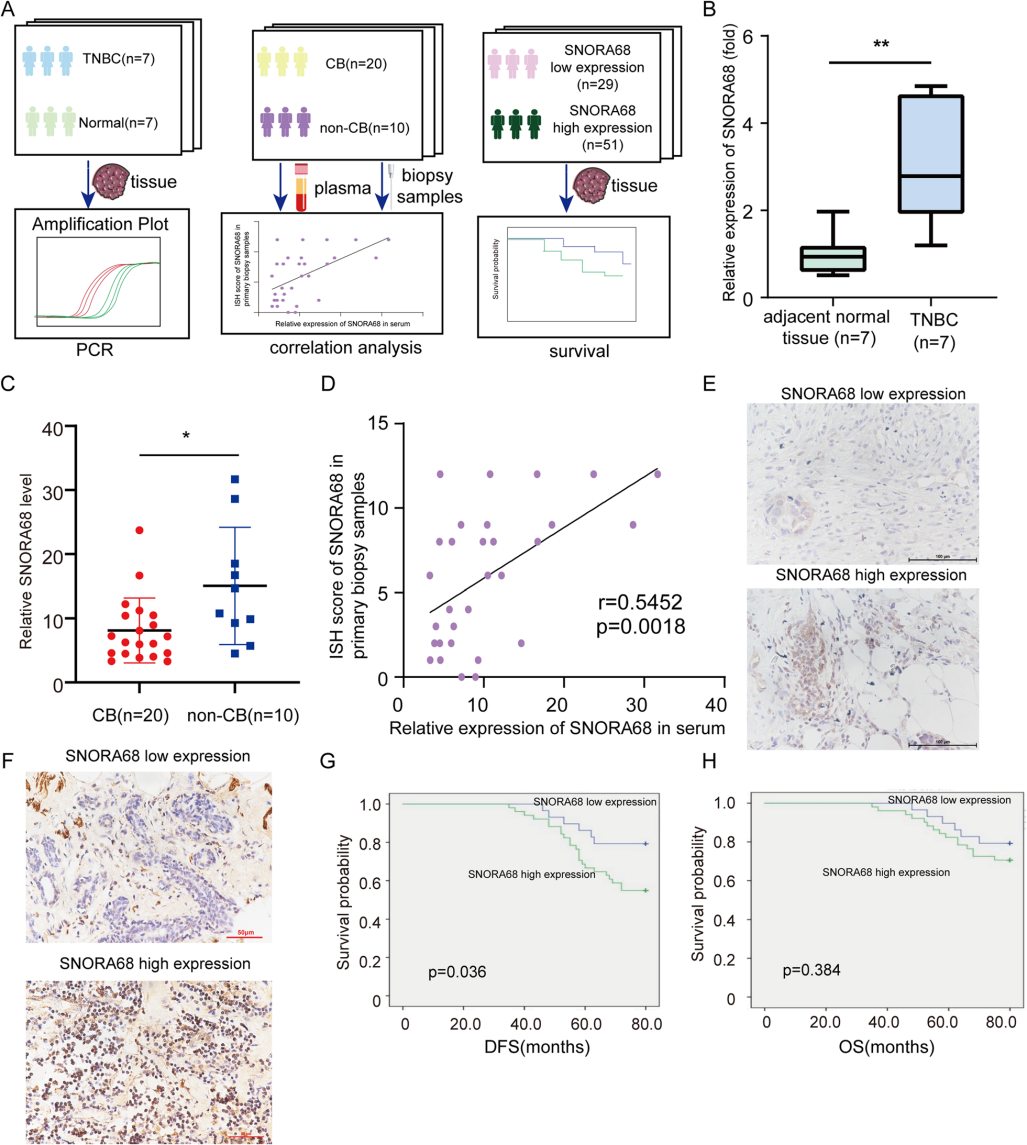
The clinical predictor SNORA68 is associated with TNBC. **A** Schematic diagram of SNORA68 as a clinical predictor of TNBC. **B** qRT-PCR was used to detect the expression of SNORA68 in TNBC tissues and normal tissues adjacent to tumors. **C** qRT-PCR was used to detect the expression of plasma SNORA68 in patients who reached CB or did not reach CB. **D** The expression of SNORA68 in primary biopsy samples was positively related to plasma SNORA68 expression. **E** Range of SNORA68 ISH in biopsy samples. **F** The expression of SNORA68 in breast cancer tissues was detected by ISH. **G**,** H** DFS and OS were compared between the SNORA68 high-expression group and the SNORA68 low-expression group. * P < 0.05, ** P < 0.01

To compare SNORA68 expression between carcinoma and adjacent tissues, 7 pairs of fresh TNBC tissues were collected. To explore the correlation between SNORA68 expression and clinicopathological factors or clinical outcomes, 80 TNBC pathological specimens were collected. To explore the correlation between SNORA68 and the efficacy of NACT, 30 biopsy samples and plasma samples from the NACT population were collected (Fig. 2A). Using qRT‒PCR, SNORA68 was found to be upregulated in fresh TNBC tissues compared to adjacent normal tissues (Fig. 2B). As previously reported, snoRNAs can stably exist in peripheral blood plasma and serum, are easily enriched and detected and can be used as potential biomarkers for liquid biopsy [9, 15]. Therefore, we detected SNORA68 in plasma and serum to explore whether SNORA68 has the potential to predict NACT efficacy. Among the 30 patients who underwent NACT, plasma SNORA68 was significantly lower in patients who reached CB than in those who failed to reach CB (8.125 versus 15.08,* P* = 0.0194) (Fig. 2C). Furthermore, this study explored the correlation between the serum SNORA68 concentration and SNORA68 expression in primary tumors. SNORA68 mRNA expression (via qRT‒PCR) was positively correlated with SNORA68 expression (via ISH) in primary biopsy samples (r = 0.5452, *P* = 0.0018; Fig. 2D, E). The expression levels of SNORA68 in 80 TNBC samples were evaluated by ISH (Fig. 2F). Higher SNORA68 expression was significantly correlated with larger tumor size (*P* = 0.048), higher ki-67 expression (*P* = 0.037) and advanced TNM stage (*P* = 0.015) (Table 1). To assess the association between SNORA68 expression and prognosis, we performed Kaplan‒Meier survival analysis and found that patients with high SNORA68 expression had significantly shorter disease-free survival (DFS) (*P* = 0.036) than patients with low SNORA68 expression (Fig. 2G). However, there was no significant difference in OS between the two groups (*P* = 0.384) (Fig. 2H).

**Table 1:** Univariate analysis of SNORA68 and clinical pathology factors

Factors	Number (%)	SNORA68 expression		χ^2^	*P-value*
		Low (%)	High (%)		
Age(years)				1.450	0.229
< 60	54 (67.5)	22 (40.74)	32 (59.26)		
> 61	26 (32.50)	7 (26.92)	19 (73.08)		
*Family history*					
No	58 (72.50)	21 (36.20)	37 (63.80)	0.000	0.990
Yes	22 (27.50)	8 (36.36)	14 (63.64)		
*Menstrual history*					
Pre-menopause	35 (43.75)	14 (40.00)	21 (60.00)	0.980	0.613
Perimenopause	9 (11.25)	4 (44.44)	5 (55.56)		
Menopause	36 (45.00)	11 (30.56)	25 (69.44)		
Tumor size(cm)				3.903	0.048*
< 2	25 (31.25)	13 (52.00)	12 (48.00)		
≥ 2	55 (68.75)	16 (29.10)	39 (70.90)		
Number of lymph node metastasis				1.335	0.513
0	32 (40.00)	12 (37.50)	20 (62.50)		
1–3	38 (47.50)	15 (39.47)	23 (60.53)		
> 3	10 (12.50)	2 (20.00)	8 (80.00)		
Histological grade				2.173	0.338
1	25 (31.25)	12 (48.00)	13 (52.00)		
2	42 (55.00)	13 (30.95)	29 (69.05)		
3	13 (16.25)	4 (30.76)	9 (69.23)		
Ki67				4.363	0.037*
< 30	32 (40.00)	16 (50.00)	16 (50.00)		
> 31	48 (60.00)	13 (27.08)	35 (72.92)		
TNM staging				8.452	0.015*
I	17 (21.25)	11 (64.71)	6 (35.29)		
II	41 (51.25)	10 (24.39)	31 (75.61)		
III	22 (27.50)	8 (36.36)	14 (63.64)		

## SNORA68 modulates tumor initiation through stemness regulation in TNBC

**Fig. 3:**
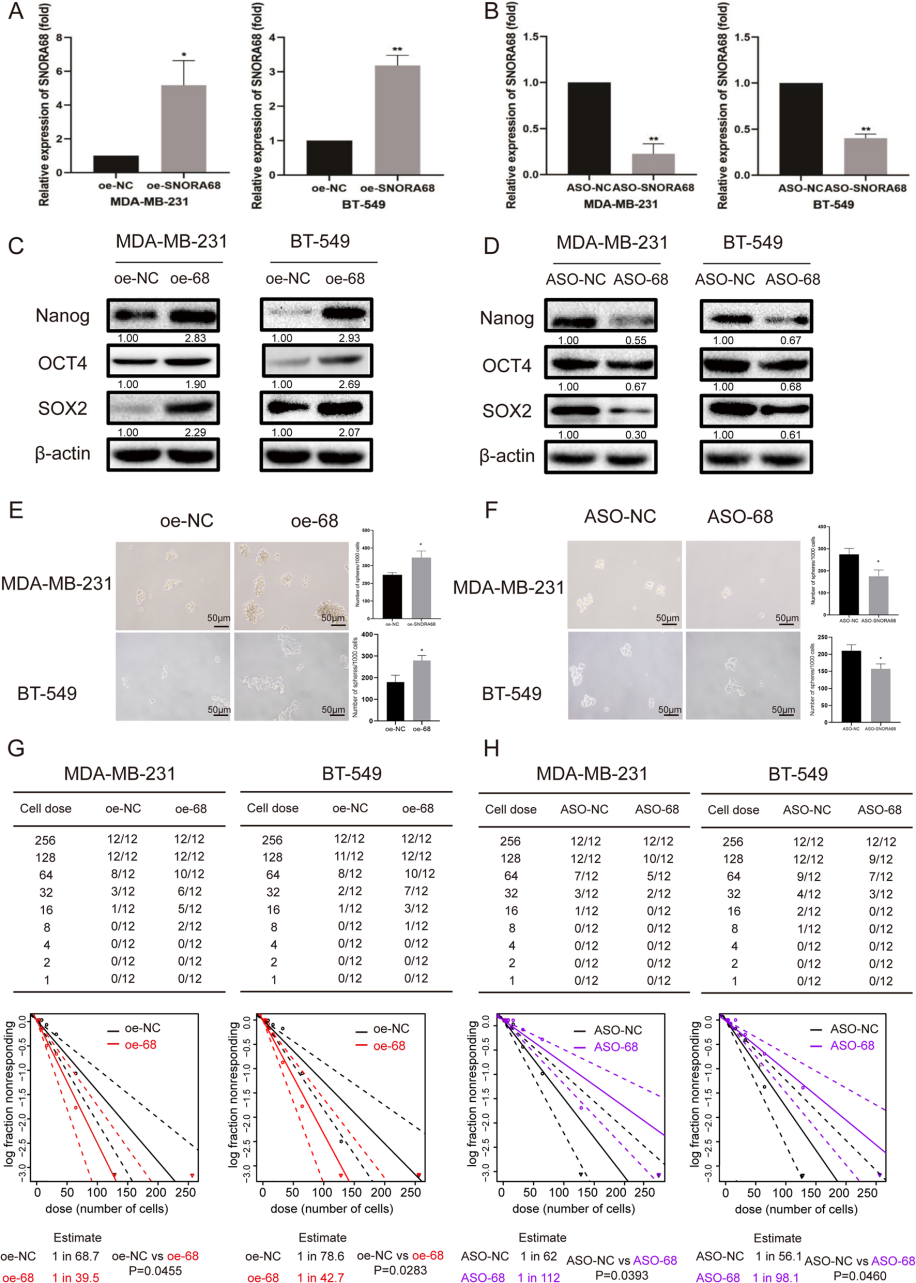
SNORA68 enhances the stemness of TNBC cells. **A** SNORA68 expression was measured by qRT-PCR in SNORA68-overexpressing MDA-MB-231 and BT-549 cells. **B** SNORA68 expression in MDA-MB-231 and BT-549 cells in which SNORA68 was silenced was measured by qRT‒PCR. **C** The expression of stemness markers (Nanog, OCT4 and SOX2) in SNORA68-overexpressing MDA-MB-231 and BT549 cells was verified by Western blot. **D** The expression of stemness markers (Nanog, OCT4 and SOX2) in SNORA68-knockdown MDA-MB-231 and BT549 cells was verified by Western blot. **E** The self-renewal ability of SNORA68-overexpressing MDA-MB-231 and BT549 cells was detected by sphere formation assays. Scale bars, 50 μm. **F** Self-renewal ability was detected in SNORA68-knockdown MDA-MB-231 and BT549 cells by sphere formation assays. Scale bars, 50 μm. **G**, **H** An in vitro LDA assay was used to determine the CSC frequency in SNORA68-knockdown MDA-MB-231 and BT549 cells. The data are presented as the mean ± SD of three independent experiments performed in triplicate. * *P* < 0.05, ** P < 0.01

To evaluate the potential biological role of SNORA68 in TNBC, we used TNBC cell lines (MDA-MB-231 and BT-549) as overexpression and knockdown models for subsequent experiments according to the SNORA68 expression level. To investigate whether SNORA68 influences the stem-like properties of TNBC cells, we constructed a SNORA68-overexpressing TNBC cell line by transfecting MDA-MB-231 and BT-549 cells with a pCMV-SNORA68 plasmid (oe-68), a SNORA68-silenced TNBC cell line and an antisense oligonucleotide (ASO-SNORA68) (Fig. 3A, B). The overexpression of SNORA68 increased the protein levels of stemness markers, including Nanog, OCT4 and SOX2, while the knockdown of SNORA68 decreased the expression of these stemness markers (Fig. 3C, D). Moreover, we determined the role of SNORA68 in the regulation of self-renewal ability and tumor initiation by conducting sphere formation and limiting dilution assays. SNORA68 increased the number of tumor spheres formed by MDA-MB-231 and BT-549 cells (Fig. 3E, F). To quantitatively assess the effect of SNORA68 on TNBC cell frequency/self-renewal, we conducted in vitro limiting dilution assays [23, 24]. As expected, fewer oe-SNORA68 TNBC cells generated spheres than oe-NC cells, while more ASO-SNORA68 TNBC cells generated spheres than ASO-NC cells (Fig. 3G, H). Taken together, our results demonstrate that SNORA68 participates in maintaining the stem cell properties of TNBC cells to attenuate tumor initiation.

## SNORA68 regulates TNBC carcinogenesis in vitro

SNORA68 overexpression significantly promoted MDA-MB-231 and BT-549 cell proliferation, whereas SNORA68 knockdown significantly suppressed TNBC cell proliferation (Additional file 1: Supplemental Figure S2A-B). Transwell assays revealed that elevated expression of SNORA68 promoted the migration of TNBC cells, while knockdown of SNORA68 decreased the migration of TNBC cells (Additional file 1: Supplemental Figure S2C). Overexpression of SNORA68 decreased the proportion of apoptotic TNBC cells, whereas downregulation of SNORA68 led to the apoptosis of TNBC cells (Additional file 1: Supplemental Figure S2D). In general, our findings showed that SNORA68 could play a key role in promoting cell proliferation and migration and repressing cell apoptosis in vitro.

## SNORA68 directly bound to U2AF2

**Fig. 4:**
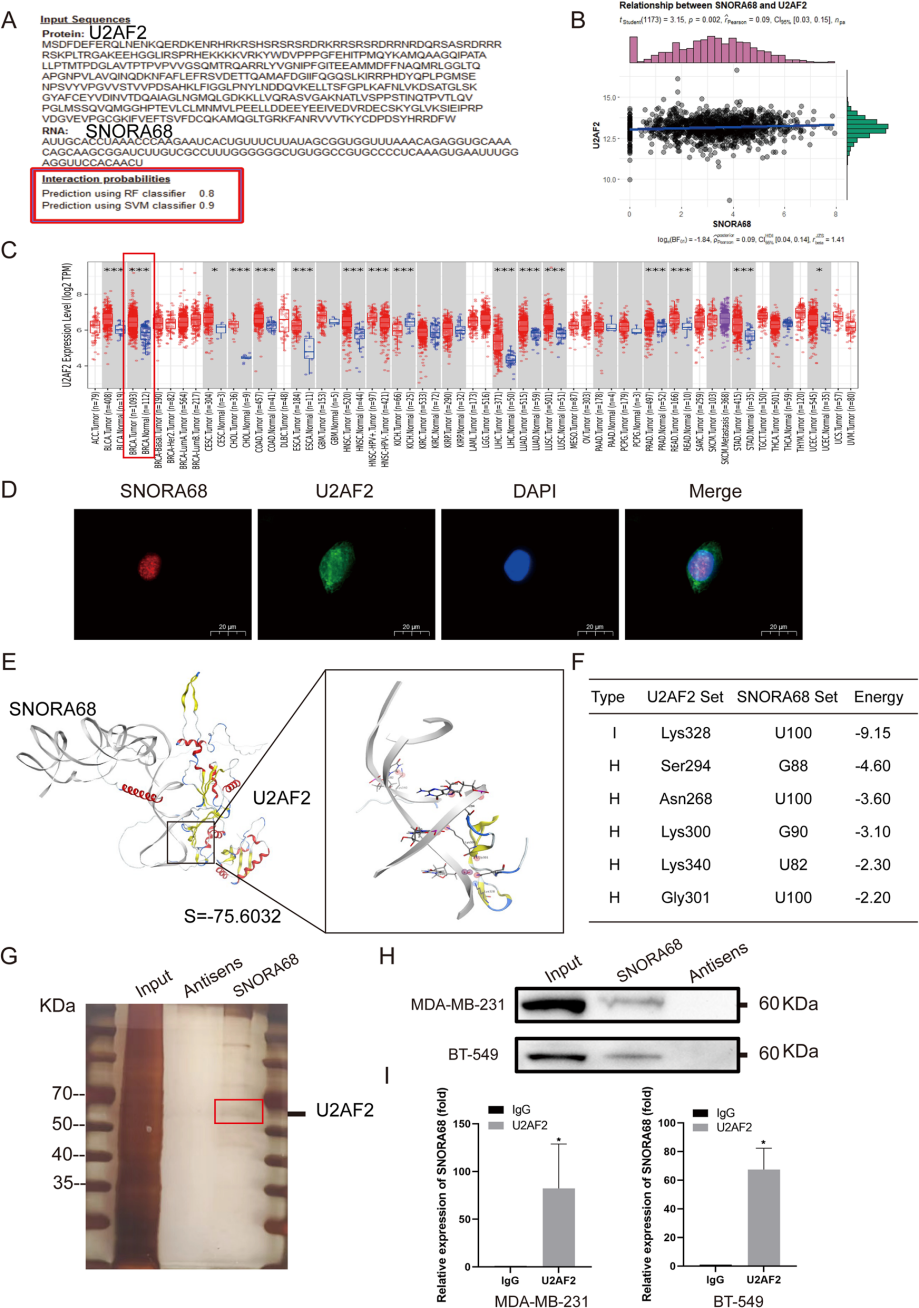
SNORA68 combines with U2AF2. **A**, **B** Bioinformatic analysis was used to predict the interaction between SNORA68 and U2AF2. **C** U2AF2 was detected by bioinformatic analysis in TCGA datasets. **D** IF and FISH were performed to determine the colocalization of SNORA68 and U2AF2 in MDA-MB-231 cells. Scale bars, 20 μm. **E**, **F** The binding sites of SNORA68 and U2AF2 were predicted by MOE software. **G** Silver staining of proteins enriched by SNORA68 compared with the negative control in MDA-MB-231 cells. **H** Western blot of U2AF2 expression in the SNORA68 pull-down precipitates in MDA-MB-231 cells. **I** qRT‒PCR analysis of SNORA68 levels in complexes enriched by RIP of U2AF2 in MDA-MB-231 and BT-549 cells. The data are presented as the mean ± SD of three independent experiments performed in triplicate. *P < 0.05, **P < 0.01

To better elucidate the molecular mechanisms of SNORA68 in TNBC, we used ENCORI and RPISeq to predict the potential proteins that bind to SNORA68 and found that U2AF2 likely binds to SNORA68 (Fig. 4A, B). U2AF2 promoted the early stage of pre-mRNA splicing by recognizing the Py tract signal [25]. U2AF2 is upregulated in various cancers but has rarely been studied in breast cancer. Bioinformatic analysis revealed that U2AF2 expression was increased in breast cancer (Fig. 4C). In addition, SNORA68 expression was positively correlated with U2AF2 in the TCGA cohort (Fig. 4C). FISH coupled with IF showed that SNORA68 and U2FA2 colocalized in the nucleus of MDA-MB-231 cells (Fig. 4D). Protein structural bioinformatics analysis with MOE software revealed an interaction between SNORA68 and U2AF2 (Fig. 4E, F). RNA pull-down assays with MDA-MB-231 extracts and mass spectrometry analysis identified potential proteins that bind specifically to SNORA68 (Fig. 4G and Additional file 1: Supplementary Table S3). Consistently, the binding of SNORA68 to U2AF2 was confirmed by independent RNA pull-down and Western blot analyses (Fig. 4H). SNORA68 was enriched in the RNA‒protein complex precipitated with an antibody against U2AF2 in MB-MDA-231 and BT549 cells (Fig. 4I). These results demonstrated that SNORA68 could directly bind to U2AF2.

## SNORA68 regulated TNBC stemness via U2AF2

**Fig. 5:**
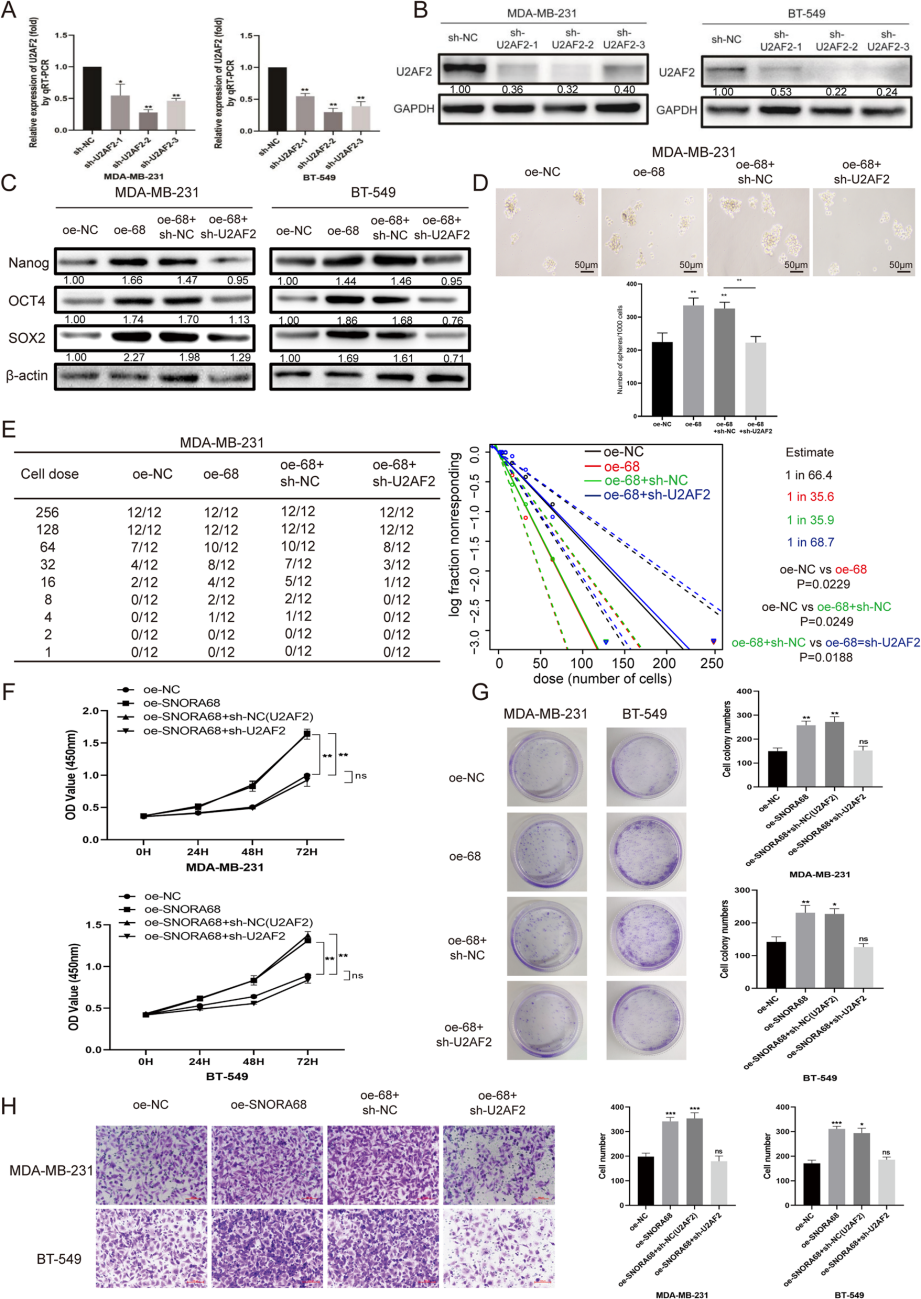
SNORA68 promotes the stemness of TNBC through U2AF2. **A** U2AF2 expression was detected by qRT-PCR in silencing U2AF2 of MDA-MB-231 and BT-549 cells. **B** U2AF2 expression was detected by Western blot in silencing U2AF2 of MDA-MB-231 and BT-549 cells. **C** The expression of stemness markers (Nanog, OCT4 and SOX2) in each group was verified by Western blot. **D** Self-renewal ability was detected in each group by sphere formation assays. Scale bars, 50 μm. **E** An in vitro LDA assay was used to determine the frequency of CSCs in each group. **F**, **G** The proliferation of TNBC cells with SNORA68 overexpression and U2AF2 knockdown was determined by CCK-8 and colony formation assays. **H** The migration of TNBC cells with SNORA68 overexpression and U2AF2 knockdown was determined by Transwell assay. Scale bars, 100 μm. The data are presented as the mean ± SD of three independent experiments performed in triplicate. * P < 0.05, ** *P* < 0.01, *** P < 0.001

To investigate whether U2AF2 mediated the function of SNORA68, we successfully generated MDA-MB-231 and BT549 cells in which U2AF2 was knocked down (Fig. 5A, B). Moreover, we overexpressed SNORA68 and silenced U2AF2 to investigate whether U2AF2 regulates TNBC tumorigenesis via SNORA68. Moreover, U2AF2 deficiency markedly reversed the increase in the expression of stemness markers (SOX2, OCT4 and Nanog) induced by SNORA68 overexpression (Fig. 5C). Sphere formation and LDA assays demonstrated that the effects of SNORA68 overexpression on the stemness of MDA-MB-231 cells could be offset by silencing U2AF2 (Fig. 5D, E). SNORA68 promoted the proliferation of TNBC cells, while U2AF2 deficiency abrogated this effect (Fig. 5F, G). Although SNORA68 overexpression promoted the migration of TNBC cells, U2AF2 knockdown reversed this effect (Fig. 5H). These results showed that U2AF2 is an essential SNORA68 protein that has an oncogenic effect on TNBC cells.

## SNORA68 binding to U2AF2 activated c-Myc signalling by retaining RPL23 in the nucleolus

**Fig. 6:**
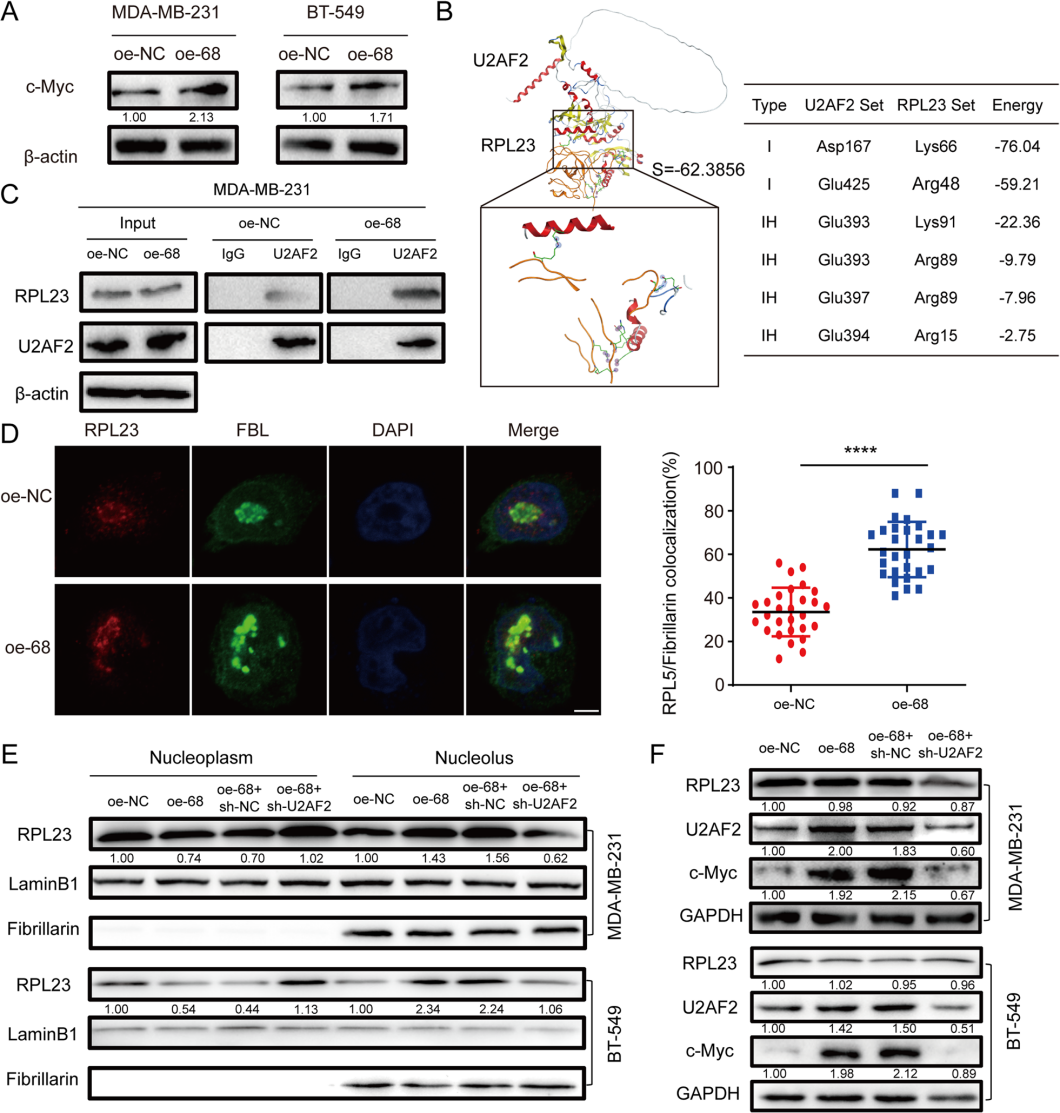
SNORA68 retains RPL23 in the nucleolus, leading to c-Myc expression via U2AF2. **A** Expression of c-Myc in SNORA68-overexpressing MDA-MB-231 and BT549 cells was verified by Western blot. **B** The binding site of RPL23 to U2AF2 was predicted by MOE software. **C** The interaction between endogenous RPL23 and U2AF2 was detected in SNORA68-overexpressing MDA-MB-231 cells by co-IP. **D** IF was performed to determine the colocalization of RPL23 in SNORA68-overexpressing MDA-MB-231 cells. Scale bars, 5 μm. **E** The expression of RPL23 in the nucleoplasm and nucleolus was measured by Western blot in different groups of TNBC cells. **F** Western blot was used to determine RPL23, U2AF2 and c-Myc expression in each group. The data are presented as the mean ± SD of three independent experiments performed in triplicate. **** *P *< 0.0001

To explore the underlying mechanism by which SNORA68 promotes the progression of TNBC, we performed enrichment analysis. GSEA revealed that gene sets, especially those related to the c-Myc signalling pathway, were positively enriched in the SNORA68 high-expression group in the TCGA dataset (Additional file 1: Supplemental Figure S3A). The overexpression of SNORA68 increased the protein level of c-Myc (Fig. 6A). We found that SNORA68 bound to U2AF2 and affected c-Myc expression. To determine how SNORA68 binds to U2AF2 to regulate c-Myc, Kyoto Encyclopedia of Genes and Genomes (KEGG) pathway enrichment analysis was performed, and the results revealed that the major enriched pathway was the ribosome pathway (Additional file 1: Supplemental Figure S3B), indicating that SNORA68 might influence ribosome stress. Surprisingly, protein structural bioinformatics analysis by MOE software indicated that U2AF2 mostly bound to RPL23 rather than RPL5 or RPL11 (Additional file 1: Supplemental Figure S3C-D and Fig. 6B). RPL23 binding to U2AF2 was greater in oe-SNORA68 cells than in control cells (Fig. 6C). Moreover, FISH coupled with IF revealed that SNORA68 retained RPL23 in the nucleus (Fig. 6D). In addition, we found that RPL23 was decreased in the nucleoplasm but increased in the nucleolus in MDA-MB-231 and BT549 cells overexpressing SNORA68, while this effect could be reversed by U2AF2 deficiency (Fig. 6E). Furthermore, SNORA68 elevated U2AF2 and c-Myc expression in TNBC cells, while U2AF2 knockdown decreased the protein expression of c-Myc (Fig. 6F). These results suggested that SNORA68 retained RPL23 in the nucleolus by binding to U2AF2 and regulating c-Myc expression.

## SNORA68 promotes TNBC tumor growth in vivo through the U2AF2/RPL23/c-Myc axis

**Fig. 7:**
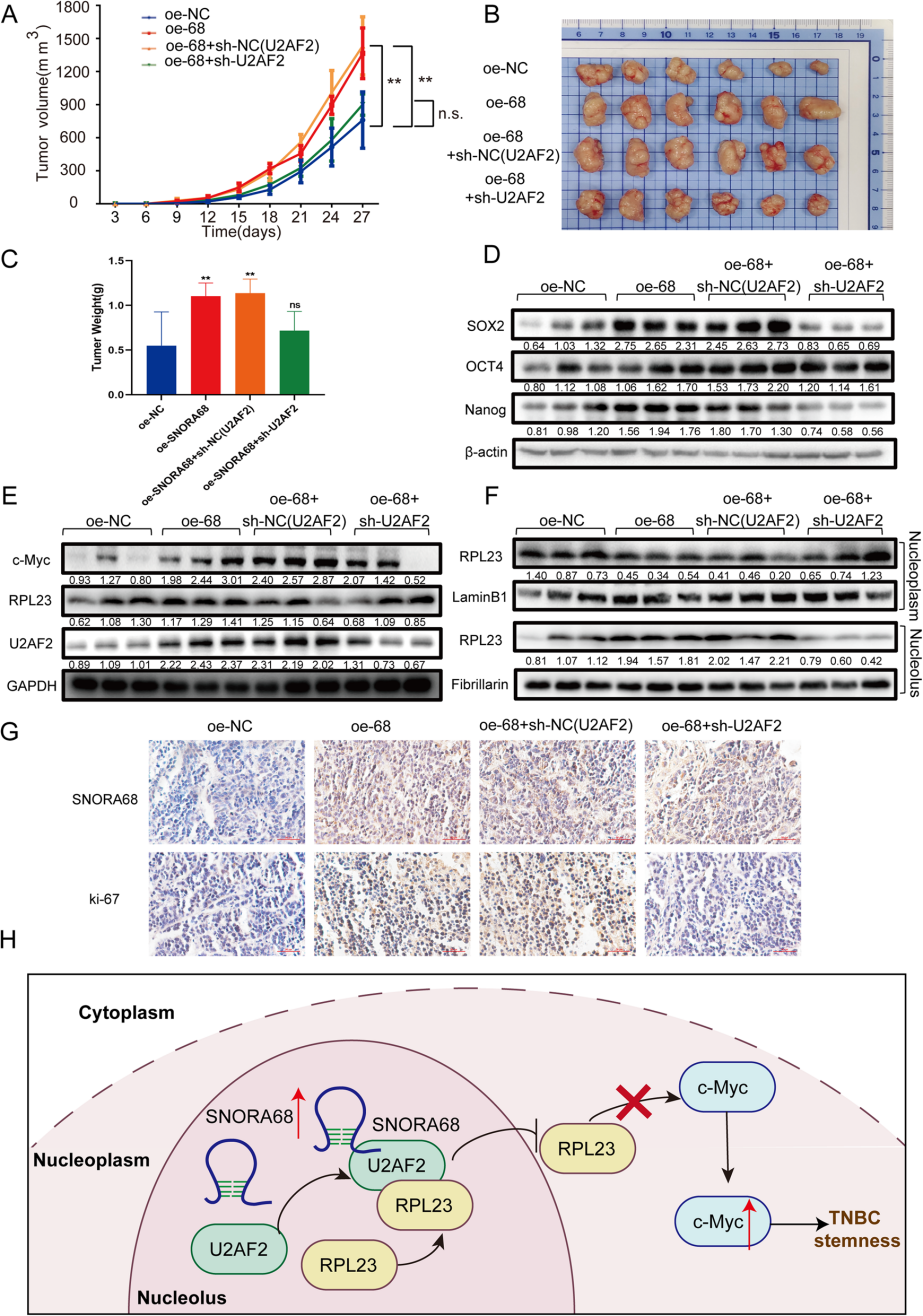
SNORA68 regulates the U2AF2/RPL23/c-Myc axis in vivo. **A** Average tumor volumes of xenograft mice were measured every three days. **B**, **C** Subcutaneous tumors and tumor weights are shown. **D** The expression of stemness markers (Nanog, OCT4 and SOX2) in the four groups was verified by Western blot. **E** Western blot was used to determine RPL23, U2AF2 and c-Myc expression in each group. **F** The expression of RPL23 in the nucleoplasm and nucleolus in the four groups of mice was measured by Western blot. **G** ISH and IHC were used to determine SNORA68 and ki67 expression. **H** Schematic of the mechanisms by which SNORA68-U2AF2 promotes TNBC stemness by retaining RPL23 in the nucleolus and reducing the RPL23-mediated decrease in c-Myc expression in the nucleoplasm. The data are presented as the mean ± SD of three independent experiments performed in triplicate. *P < 0.05, ** P < 0.01

An in vivo experiment was conducted to determine whether SNORA68 regulates TNBC tumorigenesis via the U2AF2/RPL23/c-Myc axis. Briefly, BT-549 cells were subcutaneously injected into female BALB/c nude mice to construct a xenograft model. There were 4 groups: oe-NC group (Group 1); oe-SNORA68 group (Group 2); oe-SNORA68 and sh-NC (U2AF2) group (Group 3); and oe-SNORA68 and sh-U2AF2 group (Group 4). The average tumor volume in the xenograft mice was measured every two days. After 27 days, the size of Group 2 was significantly larger than that of Group 1, while that of Group 4 was obviously smaller than that of Group 2. No significant difference in tumor volume was observed between Group 2 and Group 3 (Fig. 7A). After the isolated tumors were separated from xenograft mice, tumor weights were compared among the 4 groups, and the trends were similar to those of tumor sizes (Fig. 7B, C). Compared with those in the control group, the effect of SNORA68 on increasing tumor volume and weight was inhibited by U2AF2 knockdown. Western blot revealed that the expression of stemness markers (SOX2 and Nanog) and c-Myc in xenografts increased after SNORA68 upregulation, and this effect was reversed after cotransfection with sh-U2AF2 (Fig. 7D and E). The protein level of RPL23 in the nucleoplasm decreased, but in the nucleolus, it increased in the xenografts with SNORA68 interference and recovered after simultaneous U2AF2 knockdown (Fig. 7F). IHC and ISH assays revealed that tumors with SNORA68 overexpression exhibited increased Ki67 positivity, which was reversed in those with SNORA68 overexpression and U2AF2 loss (Fig. 7G). In summary, SNORA68 binds to U2AF2 and retains RPL23 in the nucleolus by binding U2AF2 and RPL23, reduces the decrease in c-Myc expression inhibited by RPL23 in the nucleoplasm, and ultimately promotes TNBC stemness (Fig. 7H).

## Discussion

In this study, SNORA68 was characterized by clinicopathological features and showed prognostic potential in TNBC patients. Higher SNORA68 expression was significantly correlated with a greater degree of malignancy, poorer DFS and poorer drug sensitivity to chemotherapy. As previously reported, snoRNAs can stably exist in peripheral blood plasma and serum, are easily enriched and detected and can be used as potential biomarkers for liquid biopsy [9, 15]. Therefore, we detected SNORA68 in plasma and serum to explore whether SNORA68 has the potential to predict NACT efficacy. Furthermore, this study explored the correlation between the serum SNORA68 concentration and SNORA68 expression in primary tumors. SNORA68 mRNA expression (as determined by qRT-PCR) was positively correlated with SNORA68 expression. We speculate that SNORA68 has the potential to predict NACT efficacy and may be used as a potential biomarker to indicate treatment efficacy and severity of illness for liquid biopsy. Although this is an interesting result, we performed only a preliminary analysis, and a larger sample is needed to verify these results and explore the underlying mechanism involved.

Most H/ACA box snoRNAs, in conjunction with the H/ACA snoRNP proteins DKC1, GAR1, NHP2 and NOP10, directly modify pseudouracil at specific anchor sites in eukaryotic rRNA and snRNA. This process involves converting uracil to pseudouracil and is a crucial step in ribosome biogenesis [26, 27]. It has been reported that SNORA71A promotes breast cancer metastasis by stabilizing ROCK2 mRNA through binding with the G3BP protein [11]. SNORD89 upregulation in endometrial cancer inhibits Bim and suppresses the apoptosis of endometrial cancer cells by binding to fibrillarin (Fbl) [6]. SNORD17 facilitates hepatocellular carcinoma (HCC) progression by anchoring nucleophosmin 1 (NPM1) and MYB binding protein 1a (MYBBP1A) in the nucleolus, leading to p53 inhibition [7].

We utilized an online database to identify U2AF2 as a putative binding partner for SNORA68, and subsequent RNA pull-down and RIP experiments confirmed the formation of a complex between SNORA68 and the U2AF2 protein. U2AF2 (U2 small nuclear RNA auxiliary factor 2), also known as U2AF65, is a universal splicing factor essential for pre-mRNA splicing [28]. Previous studies have linked U2AF2 to malignant progression in various tumors, including prostate cancer [29], glioma [30], and melanoma [31]. U2AF2 expression is significantly increased in lung cancer and is closely related to metastasis, advanced disease stage, recurrence and poor survival in non-small cell lung cancer patients [32]. However, its role in breast cancer remains unexplored. Through bioinformatics analysis, U2AF2 was found to be highly expressed in breast cancer, indicating a poor prognosis. Through the establishment of four cell groups and the implementation of various cell functional experiments, it was confirmed that the SNORA68 and U2AF2 proteins form a complex and collaborate to influence the self-renewal, proliferation, migration, and stemness characteristics of TNBC cells.

Bioinformatic analysis revealed that SNORA68 was closely related to the ribosomal protein pathway. Additionally, previous studies have demonstrated that SNORA23 affects liver cancer progression via the PI3K/AKT/mTOR pathway [33], while SNORA18L5 impacts the MDM2-mediated p53 pathway by influencing the binding of the ribosomal proteins L5 and L11 to MDM2 [18]. However, the detailed mechanisms underlying the effect of SNORA18L5 on ribosomal protein localization within the nucleoli remain unclear. Further exploration is needed to determine whether this effect is based on snoRNAs themselves or their interaction with proteins. Based on cell substructure isolation experiments conducted in this study, elevated expression of SNORA68 led to decreased distribution of RPL23 within the nucleoplasm but increased distribution within the nucleolus without affecting overall RPL23 protein levels. Moreover, knockdown of U2AF2 significantly reversed these effects.

Interestingly, we found that SNORA68 promoted c-Myc protein expression and that knockdown of U2AF2 significantly reversed these effects. Furthermore, co-IP assays confirmed that interference with SNORA68 overexpression reduced nuclear binding between RPL23 and U2AF2. The regulatory mechanism between RPL and c-Myc might involve complex processes. In this study, the expression of c-Myc was significantly upregulated after RPL23 entered the nucleolus, and we speculated that RPL23 might play a role in inhibiting c-Myc expression in the nucleoplasm. Based on previous studies, ribosome proteins (RPs, including RPL5, RPL11 and RPL23) bind MDM2 and bring MDM2 back to the nucleolus, thereby blocking MDM2-mediated p53 ubiquitination in the nucleoplasm and causing stable activation of p53 [34, 35]. The p53 suppresses c-Myc expression through multiple mechanisms [36].Similarly, we found SNORA68 altered the location of RPL23 in the nucleolus in this study. Therefore, we speculated that RPL23 might exert its effect through the MDM2/p53 pathway. However, the mechanism by which RPL23 regulates c-Myc expression was not further studied in this paper.

c-Myc, a crucial transcription factor, plays a pivotal role in maintaining the growth, proliferation, apoptosis, glycolysis and self-renewal of stem cells [37]. It can influence the stemness of breast cancer cells through ribosome-mediated signalling pathways [38, 39]. MYC can mediate the transcription of rRNA to promote ribosome biosynthesis, improve the efficiency of cell protein synthesis or change protein expression profiles to meet material demands for the rapid proliferation of cancer cells [40, 41]. Given the significant role of the MYC gene in cancer initiation, maintenance, and progression, targeting c-Myc represents a promising approach.

Herein, we discuss the cell lines we used. MDA-MB-231 and BT549 cells were selected for this study. First, in this study, we explored the role of SNORA68 in TNBC. MCF-7 and T47D are luminal A breast cancer cell lines, while MDA-MB-231 and BT-549 are TNBC cell lines. Therefore, we selected MDA-MB-231 and BT-549 cells for this study. Second, MDA-MB-231 and BT-549 cells are the most commonly used cell lines in breast cancer research and are widely used in most laboratories that study TNBC. Third, as shown in Fig. 1B, SNORA68 in MDA-MB-231 and BT-549 cells ranked in the middle, which was lower than that in MDA-MB-468 and HCC1937 cells and higher than that in MDA-MB-453 cells. Therefore, we selected MDA-MB-231 and BT-549 cells, which are more susceptible to overexpression and knockdown, respectively, for subsequent experiments. Fourth, we supplemented the experiment with these two cell lines. Our results showed that both SNORA68 overexpression and silencing in the MDA-MB-231 and BT-549 cell lines had stable and obvious effects.

The primary limitation lies in the lack of comprehensive analysis regarding the direct mechanism by which SNORA68 and the U2AF2 complex regulate the translocation of RPL23 from the nucleus to the cytoplasm, an aspect that warrants further investigation. The understanding of the regulatory mechanism of snoRNAs in tumors remains at a preliminary stage, leaving ample room for future exploration.

SNORA68 is a class of transactivating RNAs that function in ribosome biogenesis and, in most cases, guides the modification of preribosomal RNA. Despite length and sequence variations, SNORA68 binds to U2AF2, a pre-mRNA splicing factor that encodes a polypyrimidine (Py) tract signal in nascent transcripts. In summary, the SNORA68/ U2AF2 complex interacts with the RPL23 to induce the expression of c-Myc. This U2AF2/RPL23/c-Myc axis mediates the self-renewal, proliferation, migration, and stemness characteristics of TNBC.

### Supplementary Information

**Additional file 1**: **Figure S1.** Identification of the top differentially expressed snoRNAs between MCF-7 and MDA-MB-231. **A** qRTPCR was used to detect the expression of SNORD89 in different subtypes of breast cancer cells. **B** SNORD1 expression was measured by qRT‒PCR in different subtypes of breast cancer cells. **C** qRT-PCR was used to detect the expression of SNORA21 in breast cancer cells of different subtypes. **D** SNORD22 expression was measured by qRT‒PCR in different subtypes of breast cancer cells. **E** qRT‒PCR was used to detect the expression of SNORD99 in breast cancer cells of different subtypes. * *P* < 0.05, ** *P* < 0.01. **Figure S2.** SNORA68 promotes carcinogenesis of TNBC. **A**, **B** CCK8 and colony assays determined the proliferation of TNBC cells with SNORA68 overexpression or knockdown. **C** The migration of TNBC cells with SNORA68 overexpression or knockdown was determined by Transwell assay. Scale bars, 100 μm. **D** The apoptosis of TNBC cells with SNORA68 overexpression or knockdown was determined by flow cytometry. Data are presented as the mean ± SD of three independent experiments performed in triplicate. * *P* < 0.05, ** *P* < 0.01, *** *P* < 0.001. **Figure S3**. U2AF2 bound to SNORA68 relates to c-Myc and RPL23 expression. **A** GSEA shows that the c-Myc pathway was enriched in high SNORA68 expression. **B** KEGG enrichment analyses showed the enrichment pathways. **C** The binding site of RPL5 and U2AF2 was predicted by MOE software. **D** The binding site of RPL11 and U2AF2 was predicted by MOE software. **Table S1.** Specific protein partners of SNORA68. **Table S2.** The sequences for primers used in this study. **Table S3.** Antibodies used for IHC, IF, and WB in this study.

## Data Availability

The datasets used and/or analysed during the current study are available from the corresponding author upon reasonable request.

## Abbreviations

ASO: Antisense oligonucleotide
ATCC: American Type Culture Collection
BCSCs: Breast cancer stem cells
bFGF: Basic fibroblast growth factor
CCK-8: Cell counting kit-8
co-IP: Coimmunoprecipitation
CSC: Cancer stem cell
DAB: Diaminobenzidine
DFS: Disease-free survival
EGF: Epidermal growth factor
EMT: Epithelial–mesenchymal transition
ER: Estrogen receptor
FBS: Fetal bovine serum
FISH: Fluorescence in situ hybridization
GSEA: Gene set enrichment analysis
HCC: Hepatocellular carcinoma
HER2: Human epidermal growth factor receptor 2
IF: Immunofluorescence
IDC: Invasive ductal carcinoma
IHC: Immunohistochemistry
ISH: In situ Hybridization
KEGG: Kyoto Encyclopedia of Genes and Genomes
LDA: Limiting dilution analysis
MOE: Molecular operating environment
MS: Mammosphere
NC: Negative control
oe: Overexpression
PR: Progesterone receptor
PVDF: Polyvinylidene fluoride
qRT‒PCR: Quantitative real-time polymerase chain reaction
RPs: Ribosomal proteins
RIP: RNA-binding protein immunoprecipitation
rRNA: Ribosomal RNA
SD: Standard deviation
snoRNAs: Small nucleolar RNAs
SPF: Specific pathogen-free
TCGA-BRCA: The Cancer Genome Atlas-Breast Cancer
TNBC: Triple-negative breast cancer
U2AF2: U2 small nuclear RNA auxiliary factor 2
