# Supplementary material for: U2AF2-SNORA68 promotes triple-negative breast cancer stemness through the translocation of RPL23 from nucleoplasm to nucleolus and c-Myc expression
Source: Breast Cancer Res. 2024 Apr 9;26:60. doi: 10.1186/s13058-024-01817-6 (PMC11005140; doi:10.1186/s13058-024-01817-6)
Supplement: Supplementary file 1 — Additional file 1: Figure S1. Identification of the top differentially expressed snoRNAs between MCF-7 and MDA-MB-231. A qRTPCR was used to detect the expression of SNORD89 in different subtypes of breast cancer cells. B SNORD1 expression was measured by qRT‒PCR in different subtypes of breast cancer cells. C qRT-PCR was used to detect the expression of SNORA21 in breast cancer cells of different subtypes. D SNORD22 expression was measured by qRT‒PCR in different subtypes of breast cancer cells. E qRT‒PCR was used to detect the expression of SNORD99 in breast cancer cells of different subtypes. * P < 0.05, ** P < 0.01. Figure S2. SNORA68 promotes carcinogenesis of TNBC. A, B CCK8 and colony assays determined the proliferation of TNBC cells with SNORA68 overexpression or knockdown. C The migration of TNBC cells with SNORA68 overexpression or knockdown was determined by Transwell assay. Scale bars, 100 μm. D The apoptosis of TNBC cells with SNORA68 overexpression or knockdown was determined by flow cytometry. Data are presented as the mean ± SD of three independent experiments performed in triplicate. * P < 0.05, ** P < 0.01, *** P < 0.001. Figure S3. U2AF2 bound to SNORA68 relates to c-Myc and RPL23 expression. A GSEA shows that the c-Myc pathway was enriched in high SNORA68 expression. B KEGG enrichment analyses showed the enrichment pathways. C The binding site of RPL5 and U2AF2 was predicted by MOE software. D The binding site of RPL11 and U2AF2 was predicted by MOE software. Table S1. Specific protein partners of SNORA68. Table S2. The sequences for primers used in this study. Table S3. Antibodies used for IHC, IF, and WB in this study. [file 13058_2024_1817_MOESM1_ESM.docx]

**
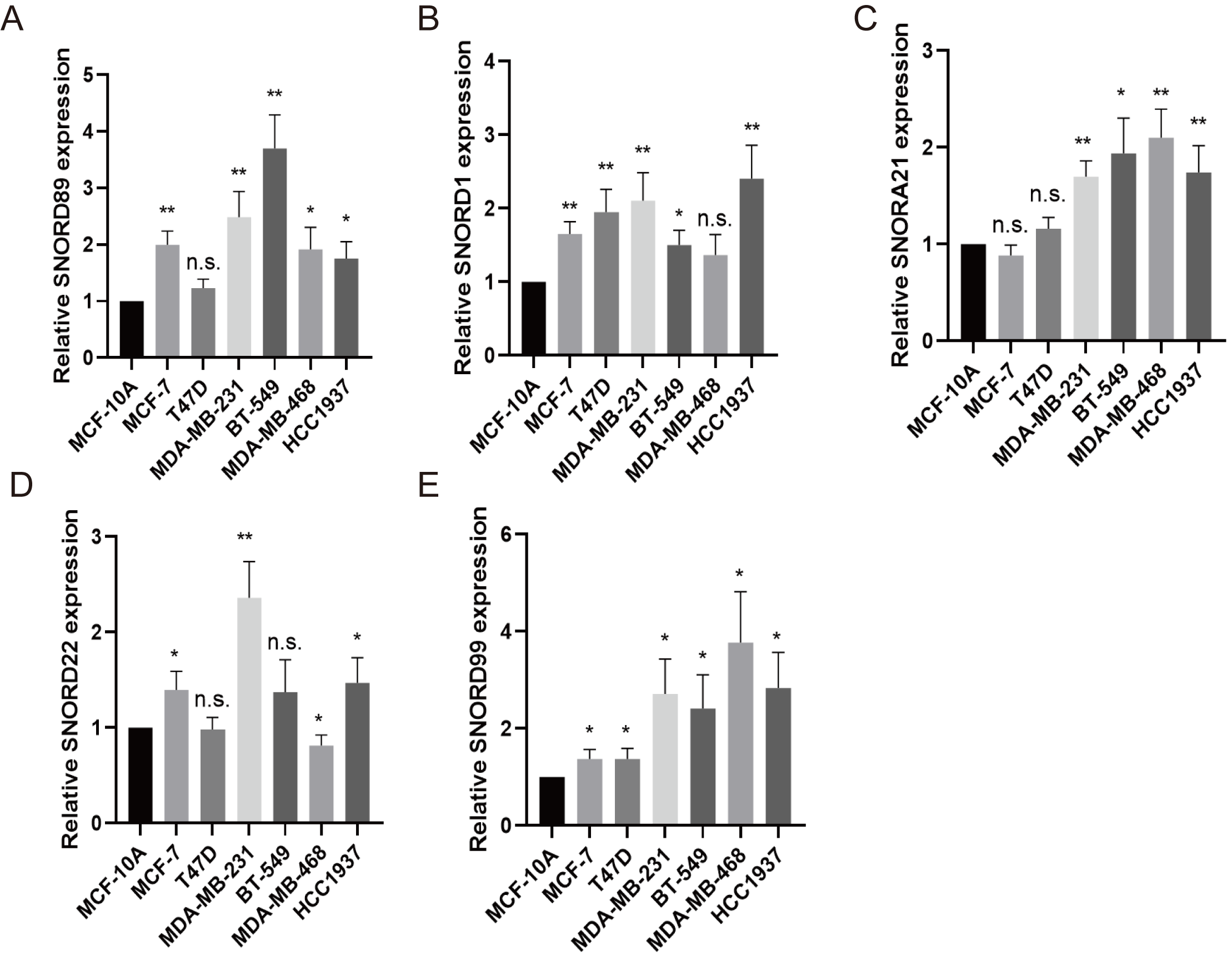
**

**Figure S1** Identification of the top differentially expressed snoRNAs between MCF-7 and MDA-MB-231 cells. (A) qRT‒PCR was used to detect the expression of SNORD89 in different subtypes of breast cancer cells. (B) SNORD1 expression was measured by qRT‒PCR in different subtypes of breast cancer cells. (C) qRT‒PCR was used to detect the expression of SNORA21 in breast cancer cells of different subtypes. (D) SNORD22 expression was measured by qRT‒PCR in different subtypes of breast cancer cells. (E) qRT‒PCR was used to detect the expression of SNORD99 in breast cancer cells of different subtypes.

**
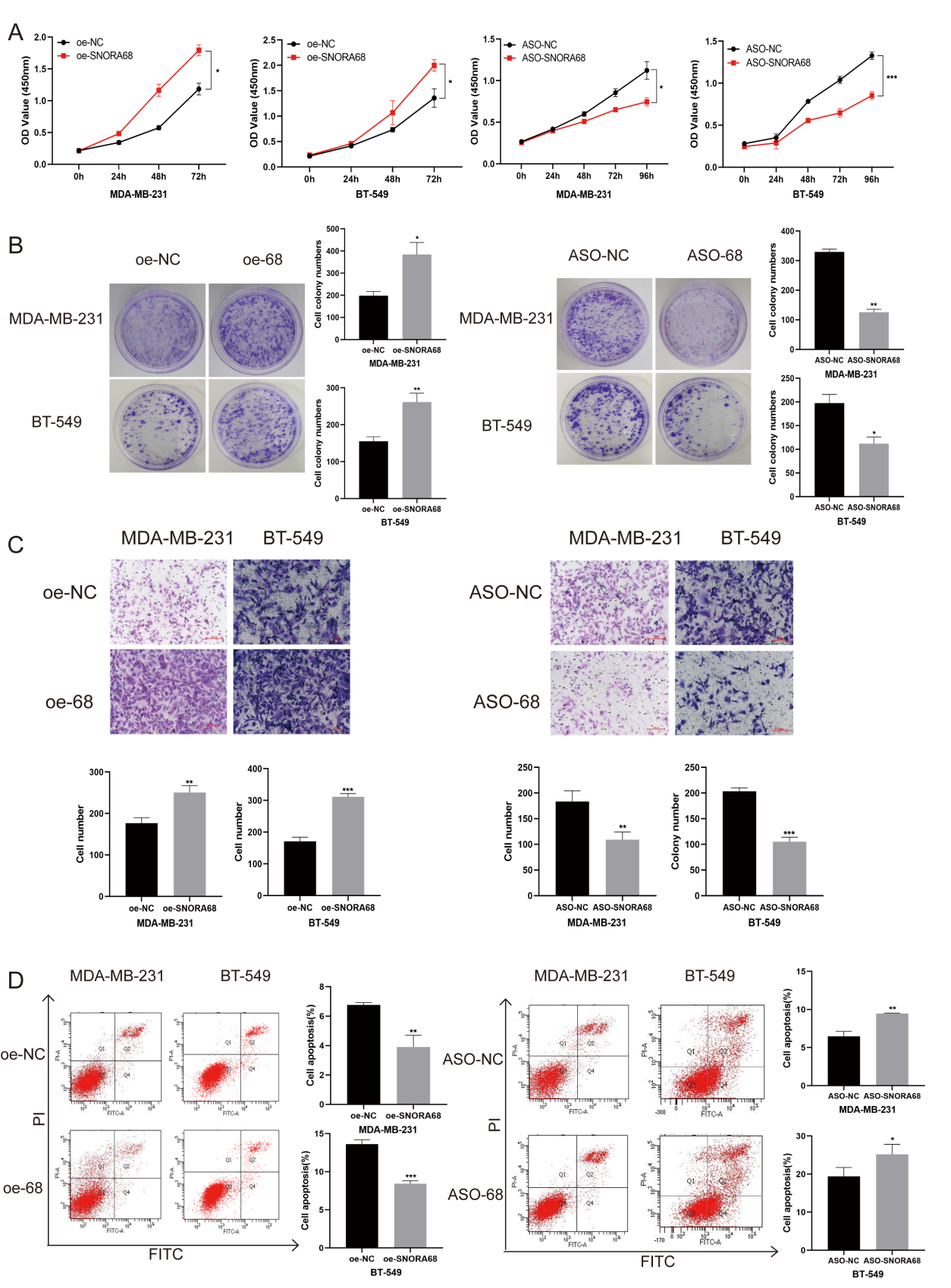
**

**Figure S2. SNORA68 promotes carcinogenesis of TNBC.** (A-B) The proliferation of TNBC cells with SNORA68 overexpression or knockdown was determined by CCK8 and colony assays. (C) The migration of TNBC cells with SNORA68 overexpression or knockdown was determined by Transwell assay. Scale bars, 100 μm. (D) The apoptosis of TNBC cells with SNORA68 overexpression or knockdown was determined by flow cytometry. Data are presented as the mean ± SD of three independent experiments performed in triplicate. *p < 0.05, **p < 0.01, ***p < 0.001, ****p < 0.0001.


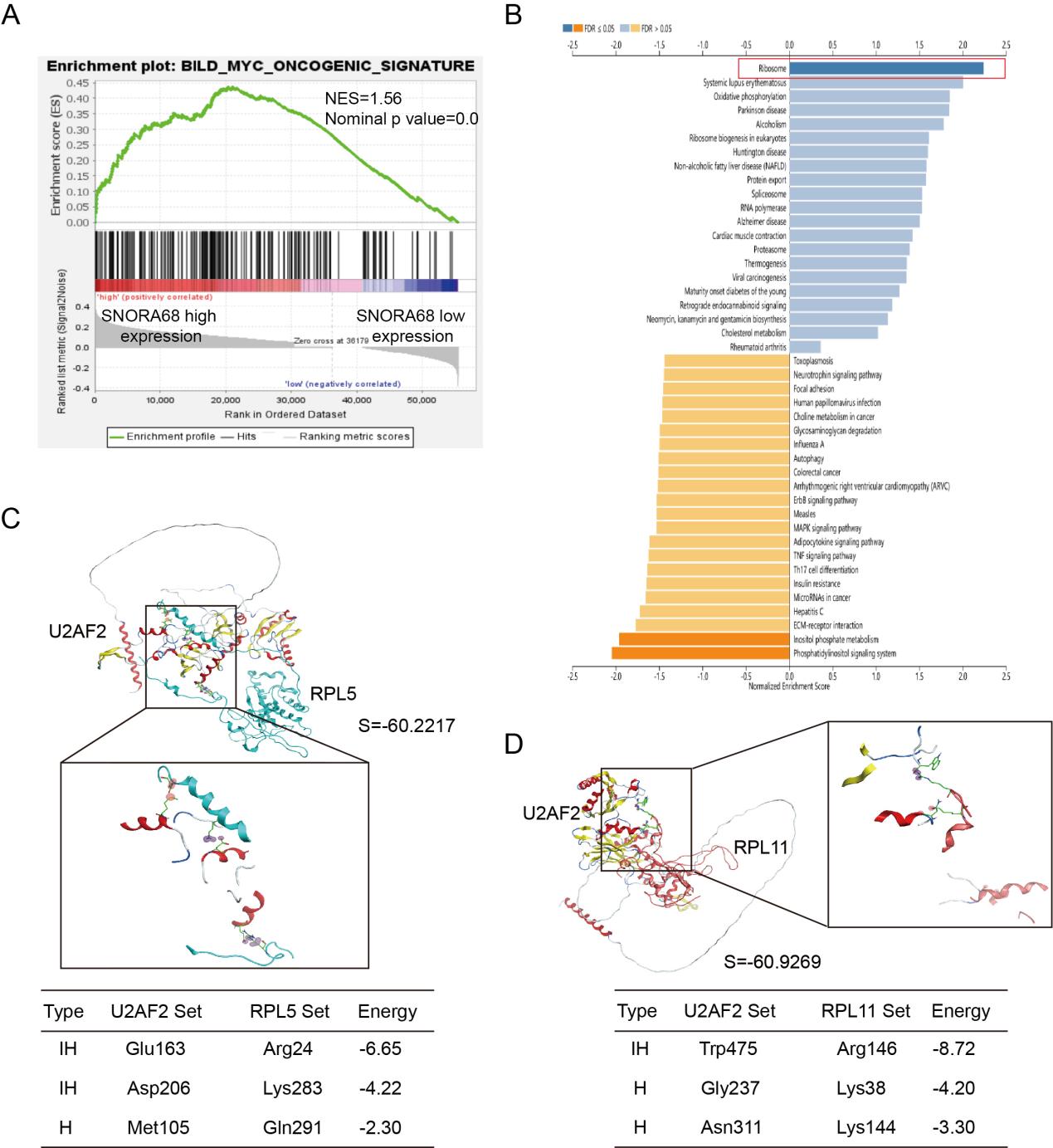


**Figure S3. U2AF2 bound to SNORA68 relates to c-Myc and RPL23 expression.** (A) GSEA shows that the c-Myc pathway was enriched in high SNORA68 expression. (B) KEGG enrichment analyses showed the enrichment pathways. (C) The binding site of RPL5 and U2AF2 was predicted by MOE software. (D) The binding site of RPL11 and U2AF2 was predicted by MOE software.

**Table S1. Specific protein partners of SNORA68**

|  |  | **SNORA68 Probes** | |  | **SNORA51 Probes** | |
| --- | --- | --- | --- | --- | --- | --- |
| **Gene names** | **Mol. weight [kDa]** | **Unique peptides** | **Intensity** |  | **Unique peptides** | **Intensity** |
| U2AF2 | 53.501 | 15 | 3026500000 |  | 0 | 0 |
| MILR1 | 38.735 | 10 | 2467480000 |  | 0 | 0 |
| EBF3 | 64.864 | 8 | 264640000 |  | 0 | 0 |
| TINAG | 54.605 | 8 | 356520000 |  | 0 | 0 |
| SAMD5 | 52.258 | 8 | 195650000 |  | 0 | 0 |
| ZNF711 | 86.245 | 7 | 204940000 |  | 0 | 0 |
| RNF125 | 26.454 | 7 | 356850000 |  | 0 | 0 |
| STRA6 | 73.503 | 7 | 206980000 |  | 0 | 0 |
| CLIC2 | 28.356 | 6 | 165894000 |  | 0 | 0 |
| TSP4 | 105.869 | 6 | 284660000 |  | 0 | 0 |
| ETFA | 35.080 | 6 | 184560000 |  | 0 | 0 |
| ZNF736 | 49.868 | 5 | 156980000 |  | 0 | 0 |
| APLN | 8.569 | 5 | 84565000 |  | 0 | 0 |
| RBM43 | 40.666 | 5 | 88562000 |  | 0 | 0 |
| SCN1A | 228.972 | 4 | 102552100 |  | 0 | 0 |
| TGM4 | 77.145 | 3 | 475610000 |  | 0 | 0 |
| CASP1 | 45.159 | 3 | 384412000 |  | 0 | 0 |
| ZNF541 | 145.587 | 3 | 798500000 |  | 0 | 0 |

**Table S2. The sequences for primers used in the study**

| **Name** | **Sequences** |
| --- | --- |
| *Primers for qRT-PCR* | |
| U6-F  U6-R | 5’-TGGCACCCAGCACAATGAA-3  5’-CTAAGTCATAGTCCGCCTAGAAGCA-3’ |
| SNORA68-F | 5’- GCTCGGATTGCCTTAGACAG-3’ |
| SNORA68-R | 5’- GGGTGAGCTTCTTGTGAGGA-3’ |
| U2AF2-F | 5’-CGGCAGCTCAACGAGAATAAA-3’ |
| U2AF2-R | 5’-GGGAACGAATCAGTCCACCG-3’ |
| β-actin -F | 5′-CTGGCCGGGACCTGACT-3′ |
| β-actin- R | 5′-TCCTTAATGTCACGCACGATTT -3′ |
| GAPDH-F  GAPDH-R | 5'-GCATCTTCTTGTGCAGTGCC-3'  5'-TACGGCCAAATCCGTTCACA-3' |

Abbreviations: qRT-PCR, Quantitative real-time PCR; ISH, in situ hybridization; F, forward primer; R, reverse primer.

**Table S3. Antibodies used for IHC, IF and WB**

| **Antibody** | **Company/Provider** | **Dilution ratio** |  |
| --- | --- | --- | --- |
| anti-human Nanog | Proteintech | 1:10000 |  |
| anti-human OCT4 | Proteintech | 1:10000 |  |
| anti-human SOX2 | Cell Signaling Technology | 1:1000 |  |
| anti-U2AF2 | Invitrogen | 1:1000 |  |
| anti-c-MYC | Proteintech | 1:10000 |  |
| anti-human RPL23 | Proteintech | 1:1000 |  |
| anti-Fibrillarin | Proteintech | 1:5000 |  |
| anti-GAPDH | abcam | 1:5000 |  |
| anti-human Lamin B1 | Cell Signaling Technology | 1:1000 |  |
| goat anti-mouse IgG | EARTHOX Life Science | 1:10000 |  |
| goat anti-rabbit IgG | EARTHOX Life Science | 1:10000 |  |
| Anti-Ki67 | abcam | 1:200 | For IHC |
| anti-U2AF2 | Invitrogen | 1:200 | For IF |
